# Supplementary material for: Identification of photoperiod-induced specific miRNAs in the adrenal glands of Sunite sheep (Ovis aries)
Source: Front Vet Sci. 2022 Jul 22;9:888207. doi: 10.3389/fvets.2022.888207 (PMC9354845; doi:10.3389/fvets.2022.888207)
Supplement: Supplementary file 1 [file Data_Sheet_1.ZIP › Supplementary file/miRDeep2 file.zip!/miRDeep2 file/Know_Structure/SPLP42A1/oar-mir-382.pdf]

miRBase precursor : oar-mir-382
Total read count : 4936
oar-miR-382-5p read count : 2138
oar-miR-382-3p read count : 2784
remaining reads : 14

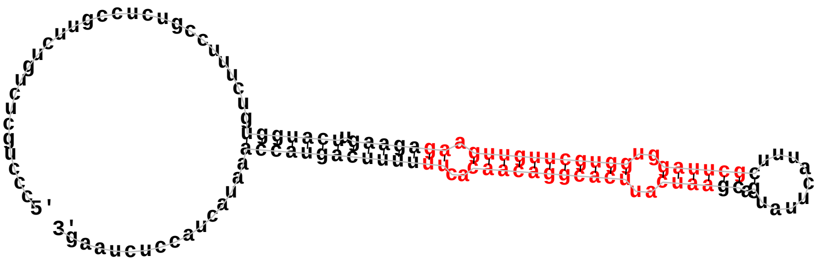

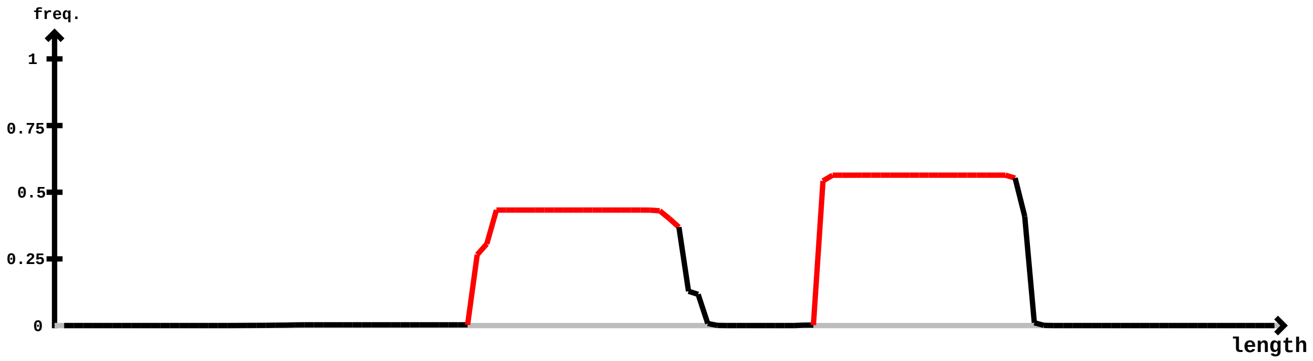


```
                                                                                 oar-miR-382-3p
                                             oar-miR-382-5p
5'- cccugcucugucuugccucugccuuucugugguacuugaagagaaguuguucgugguggauucgcuuuacuuaugacgaaucauucacggacaacacuuuuuucaguaccaaauacuaccucuaag  -3'   exp
    ....((........))............((((((.(((((((.(((((((((((..(((((((.........).))))))..)))))))))))..)))))))))))))......................   reads  mm
    .................ucugccuuucugugguacuugaaga....................................................................................   1      0
    ..................cugccuuucugugguacuugaaga....................................................................................   1      0
    ...................ugccuuucugugguacuugaaga....................................................................................   1      0
    ....................gccuuucugugguacuugaaga....................................................................................   1      0
    .....................ccuuucugugguacuugaaga....................................................................................   3      0
    ......................cuuucugugguacuugaaga....................................................................................   1      0
    .......................uuucugugguacuugaag.....................................................................................   1      0
    .......................uuucugugguacuugaaga....................................................................................   3      0
    ........................uucugugguacuugaaga....................................................................................   1      0
    ........................uucugugguacuugaagaga..................................................................................   1      0
    ........................................agaaguuguucgugguggauucg..............................................................   1      0
    ..........................................gaaguuguucgugguggau.................................................................   13     0
    ..........................................gaaguuguucgugguggauu................................................................   136    0
    ..........................................gaaguuguucgugguggauuc...............................................................   149    0
    ..........................................gaaguuguucgugguggauucg..............................................................   986    0
    ..........................................gaaguuguucgugguggauucgc.............................................................   25     0
    ...........................................aaguuguucguggugga..................................................................   1      0
    ...........................................aaguuguucgugguggauu................................................................   5      0
    ...........................................aaguuguucgugguggauuc...............................................................   11     0
    ...........................................aaguuguucgugguggauucg..............................................................   183    0
    ...........................................aaguuguucgugguggauucgc.............................................................   1      0
    ............................................aguuguucgugguggauu................................................................   2      0
    ............................................aguuguucgugguggauucg..............................................................   16     0
    ............................................aguuguucgugguggauucgc.............................................................   31     0
    ............................................aguuguucgugguggauucgcu............................................................   539    0
    ............................................aguuguucgugguggauucgcuu...........................................................   36     0
    ............................................aguuguucgugguggauucgcuuu..........................................................   3      0
    ............................................................................cgaaucauucacggacaacacu............................   1      0
    ............................................................................cgaaucauucacggacaacacuu...........................   7      0
    .............................................................................gaaucauucacggacaacacuu...........................   1      0
    ..............................................................................aaucauucacggacaacac.............................   1      0
    ..............................................................................aaucauucacggacaacacu............................   50     0
    ..............................................................................aaucauucacggacaacacuu...........................   695    0
    ..............................................................................aaucauucacggacaacacuuu..........................   1891   0
```

```
                                                                              oar-miR-382-3p
                                          oar-miR-382-5p
cccugcucugucuugccucugccuuucugugguacuugaagagaaguuguucgugguggauucgcuuuacuuaugacgaaucauucacggacaacacuuuuuucaguaccaaauacuaccucuaag

..............................................................................aaucauucacggacaacacuuuu.........................    31    0
..............................................................................aaucauucacggacaacacuuuuu........................    1    0
...............................................................................aucauucacggacaacacu............................    2    0
...............................................................................aucauucacggacaacacuu...........................    5    0
...............................................................................aucauucacggacaacacuuu..........................    78    0
...............................................................................aucauucacggacaacacuuuu.........................    17    0
...............................................................................aucauucacggacaacacuuuuu........................    3    0
..................................................................................auucacggacaacacuuu..........................    1    0
```
